# Study of Superficial Type Colorectal Neoplasms With Central Depression

**DOI:** 10.1155/DTE.6.169

**Published:** 2000

**Authors:** Sumio Fujinuma, Yoshihiro Sakai

**Affiliations:** Division of Digestive Endoscopy Ohashi Hospital Toho University School of Medicine 2-17-6 Ohashi, Meguro-ku Tokyo 153-8515 Japan

## Abstract

Superficial lesion with central depression obtained by endoscopic resection (23 carcinomas limited in the mucosa and 40 adenomas) were studied morphologically and histologically. These lesions were calculated concerning the height from the muscularis mucosa, depth of depressed central portions and the height of circumferential mucosa. Then, using the image analyzer, followings were determined with two-dimensional analysis: (1) the size of neoplasms and also (2) the size of whole mucosal lesions which was calculated by drawing a perpendicular from the border of the neoplasms; and thus, the ratio of each area was calculated.

Little difference was found between the adenomas and carcinomas. The sizes of carcinomas were found to be of 8.8 ± 4.7 mm and the adenomas of 5.1 ± 2.3 mm (*p* < 0.01). As for the depth of depression, it was found to be of 352 ± 147 μm in the carcinomas and 277 ± 93 μm in the adenomas (*p* < 0.05). Concerning the ratio of carcinomatous area in the mucosa, it was found to be 78 ± 10% in the carcinomas, while in the adenomas, it was found to be 70 ± 10% (*p* < 0.05).

Accordingly, it was found that compared with the adenomas, carcinomas showed significantly larger in size, deeper depression in configuration and the ratio of their size in the mucosa is rather high.


*Diagnostic and Therapeutic Endoscopy*, Vol. 6, pp. 169–177
Reprints available directly from the publisher
Photocopying permitted by license only

© 2000 OPA (Overseas Publishers Association) N.V.
Published by license under
the Harwood Academic Publishers imprint,
part of The Gordon and Breach Publishing Group.
Printed in Malaysia.

### Study of Superficial Type Colorectal Neoplasms with Central Depression

SUMIO FUJINUMA* and YOSHIHIRO SAKAI

*Division of Digestive Endoscopy, Ohashi Hospital, Toho University School of Medicine, 2-17-6 Ohashi, Meguro-ku, Tokyo 153-8515, Japan*

*(Received 16 November 1999; Revised 12 January 2000; In final form 16 March 2000)*

**Superficial lesion with central depression obtained by endoscopic resection (23 carcinomas limited in the mucosa and 40 adenomas) were studied morphologically and histologically. These lesions were calculated concerning the height from the muscularis mucosa, depth of depressed central portions and the height of circumferential mucosa. Then, using the image analyzer, followings were determined with two-dimensional analysis: (1) the size of neoplasms and also (2) the size of whole mucosal lesions which was calculated by drawing a perpendicular from the border of the neoplasms; and thus, the ratio of each area was calculated.**

**Little difference was found between the adenomas and carcinomas. The sizes of carcinomas were found to be of 8.8 ± 4.7 mm and the adenomas of 5.1 ± 2.3 mm ($p < 0.01$). As for the depth of depression, it was found to be of 352 ± 147 μm in the carcinomas and 277 ± 93 μm in the adenomas ($p < 0.05$). Concerning the ratio of carcinomatous area in the mucosa, it was found to be 78 ± 10% in the carcinomas, while in the adenomas, it was found to be 70 ± 10% ($p < 0.05$).**

**Accordingly, it was found that compared with the adenomas, carcinomas showed significantly larger in size, deeper depression in configuration and the ratio of their size in the mucosa is rather high.**

*Keywords:* Adenoma, Carcinoma, Central depression, Endoscopic resection, Superficial type

### INTRODUCTION

Due to the developments of the endoscopic technology and diagnosis, cases of superficial colorectal epithelial neoplasma have been detected more frequently [1,2]. While the protruding type is commonly found, the superficial type is found only 19–34% in the early stage carcinoma, and 8–46% in the adenoma [3–6]. Above all, superficial colorectal carcinoma with central depression highly tends to possess the sm invasions [1,7–11], in which case it is certainly efficient to handle with the endoscopy to search them. It is true that same appearances of adenoma is easily recognized [12–14], though, it is also important to improve the endoscopic diagnosis for the comparison between them and a certain

* Corresponding author. Tel.: +81-3-3468-1251. Fax: +81-3-3468-1269.

carcinoma or adenoma with similar macroscopic configurations. In this paper, we study the carcinoma and adenoma with the similar macroscopic shapes with materials by endoscopic resection to discuss what is the important clue in the detection of carcinoma.

### MATERIALS AND METHODS

All superficial neoplasms were obtained by endoscopic resection (ER), performed after elevating the lesions by local injection of physiological saline solution. Only specimens including the mucosa at the lesion margin were studied.

Among 1018 lesions obtained by ER during the 9-year 1-month period between July 1989 and July 1998, 324 met the histological criteria for superficial neoplasms. Sixty-three of these lesions had a central depression (40 adenoma and 23 early carcinomas) and were studied. They showed clear central depression by dye endoscopy (Figs. 1(a), 1(b), 2(a) and 2(b). All superficial neoplasms met the morphological requirements described in *General Rules for Clinical and Pathological Studies on Cancer of the Colon, Rectum, and Anus* issued by the Japanese Society for Cancer of the Colon and Rectum [15]. Lesion height was 3 mm or less. Histologically, all lesions proliferated in a horizontal direction, and none had vertically overlapping tubules. All early carcinomas were intramucosal. All carcinoma lesions were well to moderately differentiated adenocarcinomas. The lesions that penetrating the muscularis mucosa were excluded from the study following histological analysis. The histopathological diagnosis was determined with reference to the World Health Organization (WHO) classification [16]. Thus, we diagnosed the intramucosal epithelial neoplasias with an architectural abnormality with back-to-back glands as "carcinoma *in situ*" or intramucosal carcinoma. Additionally, some intramucosal tumors, whose diagnoses were not easily adopted in only the WHO's criteria for the histopathologic diagnosis, were diagnosed with reference to the morphometric atypia devised by Nakamura [17].

We studied 51 lesions obtained from men and 12 from women (mean age, men 64.2 years; women 65.5 years). Superficial neoplasms were classified according to *General Rules for Clinical and Pathological Studies on Cancer of the Colon, Rectum, and Anus* [15] as IIa (superficial elevated type, lesions that are slightly higher than the surrounding mucosa), IIb (superficial flat type, lesions with virtually no difference in height from the surrounding mucosa), or IIc (superficial depressed type,

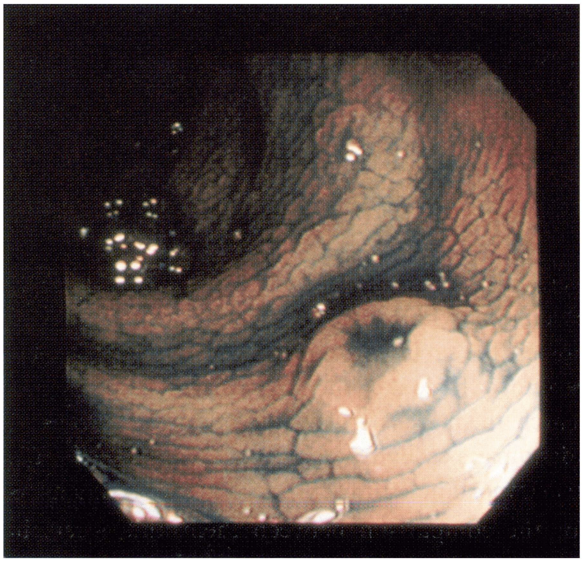

FIGURE 1(a) Endoscopic view of the adenoma.

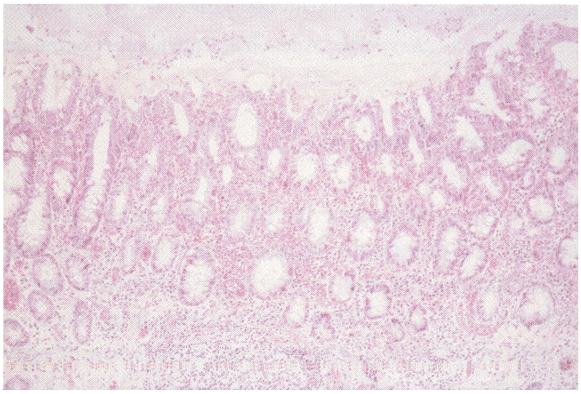

FIGURE 1(b) Microscopic view of the adenoma (HE stain, ×25).

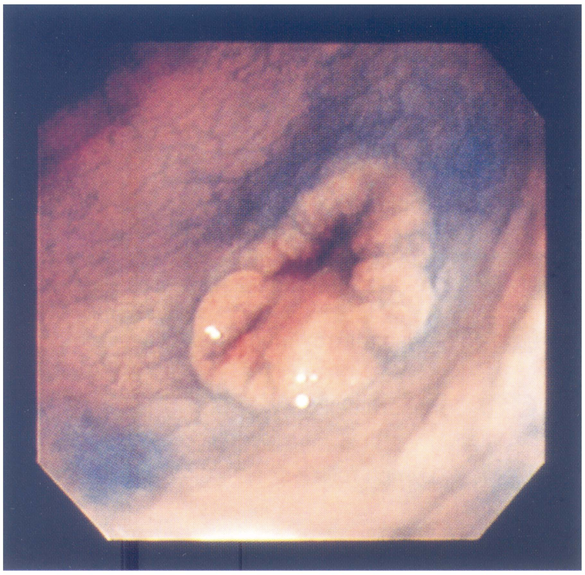

FIGURE 2(a) Endoscopic view of the early carcinoma.

lesions that are slightly lower than the surrounding mucosa). Protruding lesions with a central depression were classified as IIa + IIc. Gross lesion morphology was evaluated by referring to endoscopic findings before resection, resected specimens after they were spread out and fixed in formalin, and tissue sections prepared by conventional techniques. Any disagreement in classification was resolved by referring to tissue sections.

Endoscopically resected materials were gently spread out, pinned down, fixed in 10% formalin solution, and sliced at 3 mm intervals, taking care to include the maximum cross-sectional area of lesions with central depression. The sections were embedded in paraffin, sliced at a thickness of 4 μm, and stained with hematoxylin and eosin (HE). The lesion was first examined under a light microscope, and lesion height (A) and thickness of the surrounding mucosa as measured from the muscularis mucosae (C) were measured. Next, the depth of the depression from the crest of the lesion (B) was measured (Fig. 3). The depth of the surrounding mucosa was measured to ensure that the specimens were not excessively extended at the time of fixation. An image analyzer (Rise Co., Ltd., Tokyo, Japan)

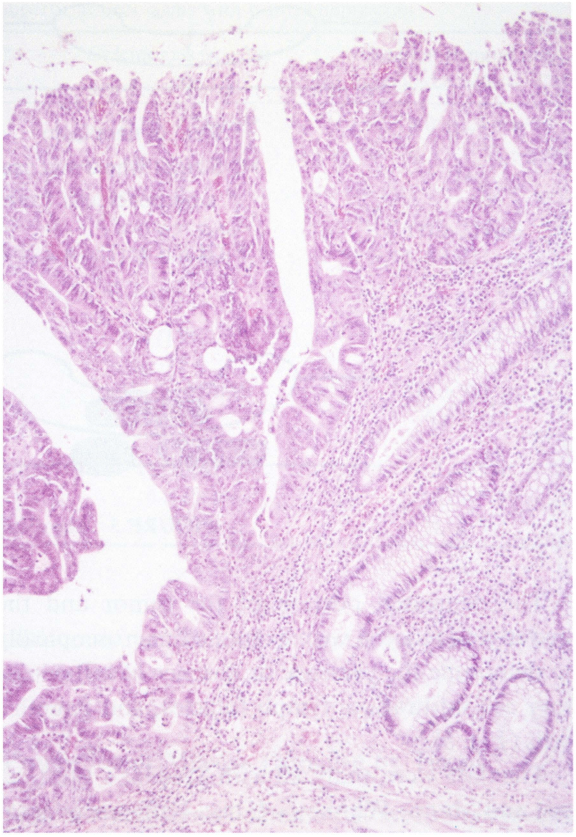

FIGURE 2(b) Microscopic view of the early carcinoma (HE stain, ×25).

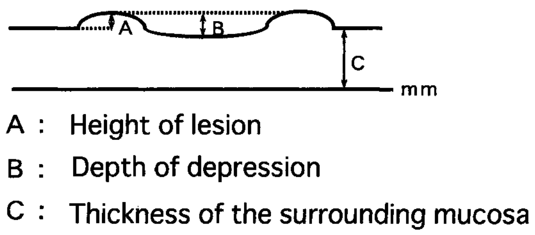

FIGURE 3 The height from the muscularis mucosa (mm), depth of central depression and the height of circumferential mucosa.

was then used to measure the area of the tumor and the area of the mucosa underlying the tumor as defined by two lines extended perpendicularly to the surface of the mucosa from the tumor border; these two variables were then used to calculate the proportion of the mucosa occupied by the tumor (Fig. 4).

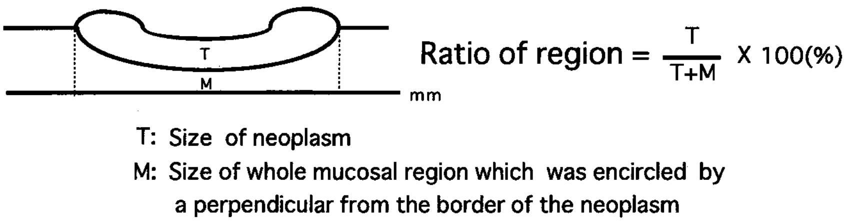

FIGURE 4 The size of neoplasms and the ratio of region.

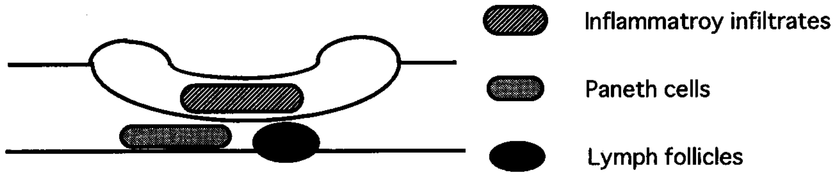

FIGURE 5 The distribution of cells in the mucosa.

The depressed portion of the tumor and the underlying mucosa were examined microscopically to determine the presence or absence of inflammatory cell infiltration, lymph follicles, and Paneth's cells (Fig. 5).

The data were analyzed with Student's *t*-test, $\chi^2$ test and Mann–Whitney's *U* test. Differences associated with $p < 0.05$ were regarded as significant.

### RESULTS

### Tumor Size and Site

Among 40 adenomas studied, 15 were located in the sigmoid colon, 9 in the descending colon, 14 in the transverse colon, and 2 in the ascending colon. Of 23 carcinomas studied, 3 were located in the rectum, 6 in the sigmoid colon, 3 in the descending colon, 7 in the transverse colon, and 4 in the ascending colon. There was no distinct difference in location between adenomas and carcinomas. The mean tumor diameter was $5.1 \pm 2.3$ mm for the 40 adenomas and $8.8 \pm 4.7$ for the 23 carcinomas. The carcinomas were significantly larger than the adenomas. The 16 adenomas measuring 5 mm or more in diameter were similar in size to the carcinomas. Therefore, the morphologic characteristics of all adenomas ($n = 40$) and adenomas of 5 mm or more in diameter ($n = 16$) were compared with those of the carcinomas (Table I).

### Macroscopic Classification of Depression

Depression was divided into IIa + IIc and IIc and compared between the adenomas and carcinomas. We classified the macroscopic type of depression as IIa + IIc, IIc, and IIc + IIa according to the *General Rules for Clinical and Pathological Studies on Cancer of the Colon, Rectum, and Anus* issued by the Japanese Society for Cancer of the Colon and Rectum [15]. Among the 40 adenomas, there were 34 IIa + IIc lesions and 6 IIc lesions. The 23 carcinomas consisted of 21 IIa + IIc lesions and 2 IIc lesions. There was no significant difference between these lesions. All 16 adenomas 5 mm or more in diameter were IIa + IIc lesions. There was no difference as compared with the carcinomas (Table I).

### Lesion Height and Thickness of Surrounding Mucosa

Lesion height and the thickness of the surrounding mucosa were compared between the adenomas and carcinomas (Fig. 3). Mean thickness of the

TABLE I Clinicopathological characteristics of superficial neoplasms with central depression

| | Adenomas ($n = 40$) | Carcinomas ($n = 23$) | Adenomas more than 5 mm in diameter ($n = 16$) |
|---|---|---|---|
| Size | 5.1 ± 2.3 mm** | 8.8 ± 4.7 mm** | 8.0 ± 1.9 mm |
| Macroscopic classification | | | |
| IIa + IIc | 34 | 21 | 16 |
| IIc | 6 | 2 | 0 |
| Height (A) | 340 ± 211 μm | 430 ± 275 μm | 437 ± 239 μm |
| Thickness of the surrounding mucosa (C) | 535 ± 98 μm | 573 ± 84 μm | 550 ± 111 μm |
| Depression compared with surface of the surrounding mucosa | | | |
| Absolute | 8 (18%) | 4 (17%) | 3 (18%) |
| Relative | 32 | 19 | 13 |
| Depth of depression (B) | 277 ± 93 μm* | 352 ± 147 μm* | 318 ± 95 μm* |
| Ratio of neoplasm (T/T + M) | 70 ± 10%* | 78 ± 10%* | 70 ± 10%* |
| The distribution of cells in the mucosa | | | |
| Inflammatory infiltrates | 14 (35%) | 12 (52%)* | 3 (18%)* |
| Paneth cells | 4 (10%) | 4 (17%)* | 9 (56%)* |
| Lymph follicles | 29 (73%) | 13 (57%) | 13 (81%) |

**$p < 0.01$; *$p < 0.05$.

surrounding mucosa was similar for adenomas (535 ± 98 μm) and carcinomas (573 ± 84 μm). Although both tumor types were similar with respect to the morphometric variables studied, lesion height was slightly higher for carcinomas (430 ± 275 μm) than for all adenomas (340 ± 211 μm), but similar to that of adenomas measuring 5 mm or more (437 ± 239 μm) (Table I).

### Relative Depression and Absolute Depression

Lesions were classified according to whether they had relative depression (i.e., the base of the depression was higher than the surface of the mucosa) or absolute depression (i.e., the base of the depression was lower than the surface of the mucosa). Absolute depression was present in similar proportions of adenomas (8 of 40, 20%) and carcinomas (4 of 23, 17%). There was also no difference between the adenomas measuring 5 mm or more (3 of 16, 18%) and carcinomas (Table I).

### Depression Depth

Depression depth (Fig. 3) was significantly higher for carcinomas (352 ± 147 μm) than for adenomas (277 ± 93 μm), but similar for carcinomas and adenomas measuring 5 mm or more (318 ± 95 μm) (Table I).

### Proportion of Mucosa Occupied by Tumor

Since adenomas and carcinomas were similar with respect to lesion height and depth, the two-dimensional proportion of the mucosa occupied by tumor was estimated with an imaging analyzer (Fig. 4). The area of the mucosa occupied by tumor was 70 ± 10% for all adenomas, 78 ± 10% for carcinomas, and 70 ± 10% for adenomas measuring 5 mm or more. These values were significant (Table I).

### Interstitial Changes in the Mucosa

Chronic inflammatory cell infiltration of moderate or higher severity, Paneth's cells and lymph follicles were evaluated as indices of interstitial changes (Fig. 5). Chronic inflammatory cell infiltration of moderate or higher severity was found in similar proportions of all adenomas (14 of 40, 35%) and carcinomas (12 of 23, 52%). This was significantly higher between the carcinomas and adenomas measuring 5 mm or more (3 of 16, 18%). Paneth's cells were present in similar proportions of all adenomas

(4 of 40, 10%) and carcinomas (4 of 23, 17%). This was significantly lower between carcinomas and adenomas measuring 5 mm or more (9 of 16, 56%). Lymph follicles were present in similar proportions of all adenomas (29 of 40, 73%), carcinomas (13 of 23, 57%), and adenomas measuring 5 mm or more (13 of 16, 81%) (Table I).

### DISCUSSION

Increasing numbers of superficial colorectal carcinomas have been detected recently [18–20]. These lesions have led to new theories for the development and spread of colorectal neoplasms. Although early colorectal carcinomas were once considered to consist primarily of protruding type lesions, increasing numbers of depressed type intramucosal carcinomas have been reported since the 1980s. Subsequently, a growing body of evidence has suggested that superficial colorectal carcinomas showing a central depression rapidly invade the submucosa and develop into advanced cancers [7–11,21]. The majority of superficial colorectal neoplasms are adenomas rather than carcinomas, and many depressed type adenomas have been detected [12–14,22]. It is important to compare these lesions with intramucosal carcinomas showing a depression. The pattern of the surface mucosa of intramucosal carcinomas has been examined by magnifying endoscopy or under a dissecting microscope, and lesions have been classified on the basis of their pit pattern or imaging characteristics [23,24]. We considered it necessary to study the perpendicular development of depressed lesions and compared their morphologic characteristics with those of superficial colorectal lesions, including the surrounding mucosa, obtained by ER.

Adenomas and carcinomas were located principally in the sigmoid colon, and there was no difference in their distribution in the colon or rectum. The location of these lesions was similar to adenomas and carcinomas with no depression. Lesion depression was not associated with a difference in location. However, the diameter of carcinomas (8.8 ± 4.7 mm) was significantly larger than that of adenomas (5.1 ± 2.3 mm). Given that other macroscopic characteristics are similar, whether increased lesion size is associated with the presence of carcinoma or whether carcinoma is present regardless of lesion size should be compared between adenomas and carcinomas. When only adenomas of 5 mm or more in diameter were studied, we found that their size (8.0 ± 1.9 mm) was similar to that of carcinomas, indicating that size-related factors can be disregarded. We, therefore, compared the characteristics of carcinomas with those of all adenomas as well as with those of adenomas measuring 5 mm or more.

We classified the macroscopic type of depression as IIa + IIc, IIc, and IIc + IIa according to the *General Rules for Clinical and Pathological Studies on Cancer of the Colon, Rectum, and Anus* issued by the Japanese Society for Cancer of the Colon and Rectum [15]. There were no IIc + IIa lesions in the study groups. The numbers of IIa + IIc lesions were similar for all adenomas (34 and 6, respectively) and for carcinomas (21 and 2). Similar results were obtained for adenomas measuring 5 mm or more. Therefore, for lesions with a IIc region, the concurrent presence or absence of a IIa region does not indicate an increased risk of cancer. When adenomas and carcinomas were combined, IIa + IIc lesions (55 of 63 lesions, 87%) greatly outnumbered IIc lesions; lesions with only a central depression were rare. This is consistent with the fact that most lesions with a central depression first appear as protruding lesions on endoscopic examination, and the depression becomes distinct after the application of dyeing with methylene blue.

The absolute height of lesions cannot be measured on fixed specimens. Fresh specimens contract slightly after fixation in formalin, and lesion size is affected by uneven tension applied to the specimen when it is pinned in place [25]. To adjust for these changes in size, we measured the thickness of the normal mucosa surrounding the lesion. The thickness of the normal mucosa decreases slightly with age, but this does not affect the measurement of lesion size. Therefore, if the thickness of the surrounding mucosa is generally stable, the measured value including lesion height is considered reliable. Lesion height was slightly higher for carcinomas (430 ± 275 μm) than for all adenomas (340 ± 211 μm), but similar for adenomas measuring 5 mm or more (437 ± 239 μm) and carcinomas. These findings indicate that lesion height increases as lesions grow horizontally, with no apparent difference between adenomas and carcinomas. Tsuruta *et al.* also measured 16 cases of superficial colorectal carcinoma with central depression to report that the height of the lesions: 776 ± 339 μm/height of the circumferential mucosa: 396 ± 99 μm [5]. However, their definition on the height could be the distance from the muscularis mucosa, it is inevitable to admit the slight difference from our results: 380 ± 240 μm. The difference of the mean size of Tsuruta's from our figure; 430 ± 275 μm, should be caused by the studied lesions' size. It is natural to consider that they approved of the results from rather smaller lesions than ours.

The clinical significance of lesions detected on endoscopic examination may differ according to the classification of relative depression and absolute depression [26]. The proportion of lesions with absolute depression was similar for all adenomas (8 of 40 lesions, 20%), carcinomas (4 of 23 lesions, 17%), and adenomas of 5 mm or more in diameter (3 of 16 lesions, 18%). These results indicate that absolute depression was not useful in distinguishing adenomas from carcinomas, because about 20% of both types of lesions had absolute depression. Also, according to the statement by Tsuda *et al.*, there should be no differences in either relative or absolute depressions [25].

The depth of relative depression may be useful in the endoscopic evaluation of lesions. We, therefore, measured the depth of depression. The depth of depression was significantly greater for carcinomas (352 ± 147 μm) than for all adenomas (277 ± 93 μm), but there was no difference between adenomas measuring 5 mm or more (318 ± 95 μm) and carcinomas. This indicates merely that the depth of depression parallels lesion size for both adenomas and carcinomas; it does not imply that depression tends to form in the presence of carcinoma. In other words, a central depression becomes more easily recognizable on endoscopic examination of large lesions.

The proportion of the mucosa occupied by tumor was estimated on maximal-area sections by means of a two-dimensional image analyzer. Intramucosal neoplastic lesions range from tumors present only in the superficial layer to those that replace the entire mucosa. We previously classified intramucosal lesions invading only the surface layer of the mucosa as $m_1$, those invading all layers of the mucosa as $m_3$, and those showing intermediate invasion as $m_2$ [19]. In the present study, however, we used two-dimensional image analysis to objectively quantify the extent of the mucosa occupied by tumor. This technique clearly distinguishes the tumor tissue in the mucosa from normal tissue; moreover, the area of the mucosa occupied by tumor can be expressed as a ratio. Our results showed that the proportion of the mucosa occupied by tumor was significant for all adenomas (70 ± 10%), carcinomas (78 ± 10%), and adenomas of 5 mm or more in diameter (70 ± 10%). Although the proportion of the mucosa occupied by tumor was significantly higher for carcinomas. Wada *et al.* observed that total diameter of the depressed region of the lesion and the maximum diameter of the entire lesion were measured using the computed image analyzer and were used to compute the percent depressed region of similar intramucosal carcinomas [27]. They reported that a lesion of less than 5 mm in diameter was significantly higher in the mean percent depressed region of carcinoma than a lesion of greater than 6 mm in diameter. Accordingly they reported that those lesions with a larger diameter or deeper invasion revealed lower percent depressed regions in each lesions. It was different from our data. Because we studied the proportion of the mucosa occupied by tumor, not the diameter of the depressed region of tumor. Quite few studies have been reported regarding the proportion of mucosa occupied by tumor, yet, it could be positive steps to suggest the further research of cancer invasion process. On the process of cancer invasion in the mucosa, it might be growing vertically to displace normal tubules then the larger the proportion of the tumor-occupied mucosa is getting, the deeper the depression is.

Because the superficial depressed neoplasms showed carcinoma at the average of 8.8 mm in diameter while adenoma at the average of 5.1 mm, it suggest that the carcinogenesis be related with gaining in size and the depression depth.

We focused on structural elements of neoplasms, such as ducts, stroma, and vessels. Vessels include capillaries and lymphatic ducts in the mucosa, but these elements are unlikely to affect the macroscopic characteristics of tumors. We thus compared adenomas and carcinomas with regard to the presence of chronic inflammatory cell infiltration of moderate or higher severity, Paneth's cells and lymph follicles. Chronic inflammatory cell infiltration, Paneth's cells and lymph follicles were considered indices of past inflammation, present inflammation, and secondary evidence related to the central depression, respectively. There was no difference in these indices between adenomas and carcinomas. However, concerning the inflammatory infiltrates and appearance of Paneth cells showed significantly high in the adenomas more than 5 mm in diameter and carcinomas. Interstitial changes were, therefore, considered secondary findings related to lesion size and did not differ between adenomas and carcinomas.

Endoscopically, superficial depressed colorectal neoplasms appeared as plaque-like lesions with vague redness or discoloration. Kudo *et al.* reported that the key point in colonoscopic observation is to detect a change of color, that is, slight redness and pallid mucosa [6]. Hayakawa *et al.* reported that depressions became evident only after indigo carmine spraying in 26 of the 50 lesions (52%) [28]. Furthermore, Jaramillo *et al.* reported that these lesions were detected by high-resolution video endoscopy and chromoscopy [29]. We have used the dyeing with methylene blue to detect these lesions. Both indigo carmine and methylene blue spraying are useful to detect for these lesions. Therefore, the endoscopic detection for the superficial carcinoma with central depression requires the necessity to find the large depression of lesion more than 5 mm in diameter using any kind of dyeing method.

### CONCLUSIONS

We endoscopically resected superficial colorectal neoplasms that had a central depression and compared the characteristics of adenomas with those of carcinomas. The following results were obtained. (1) Carcinomas ($8.8 \pm 4.7$ mm) were significantly larger than adenomas ($5.1 \pm 2.3$ mm). (2) There was no difference between adenomas and carcinomas in macroscopic classification and lesion height. (3) Depression height was more significant in carcinomas than in adenomas. (4) The proportion of mucosa occupied by tumor was significantly larger for carcinomas ($78 \pm 10\%$) than for adenomas ($70 \pm 10\%$). (5) Although there was no difference between adenomas and carcinomas in interstitial changes such as chronic inflammatory cell infiltration of moderate or above, Paneth's cells or lymph follicles. Interstitial changes were considered secondary changes related to lesion size rather than to either adenomas or carcinomas.

Therefore, the endoscopic detection for the superficial carcinoma with central depression requires the necessity to find the large depression of lesion more than 5 mm in diameter using any kind of dyeing method.

### *References*

[1] Nagasako, K., Tanaka, Y., Ohara, N. *et al.* System for the diagnosis of superficial early colorectal cancer. *Gastroenterol. Surg.* 1991; **14**: 307–315.

[2] Kudo, S., Tamura, S., Hirota, S. *et al.* The problem of *de novo* colorectal carcinoma. *Eur. J. Cancer* 1995; **31**: 1118–1120.

[3] Ohkura, Y. Clinicopathological features of superficial colorectal carcinomas. *Jpn. J. Clin. Radiol.* 1995; **40**: 1233–1242.

[4] Kudo, S., Nakajima, K., Kusaka, N. *et al.* Macroscopic classification of minute colorectal tumor. *Stomac. Intest.* 1994; **29**: 27–38.

[5] Tsuruta, O., Arima, N., Toyonaga, J. *et al.* Macroscopic classification of minute colorectal tumor: from endoscopic viewpoint. *Stomac. Intest.* 1994; **29**: 37–41.

[6] Kudo, S., Tmura, S., Nakajima, T. *et al.* Depressed type of colorectal cancer. *Endoscopy* 1995; **27**: 54–57.

[7] Wada, R., Abe, H., Hirai, S. *et al.* Pathological findings and the cell cycles of minute adenomas and adenocarcinomas of the depressed type in the large bowel. *Jn. J. Cancer Clin.* 1992; **38**: 33–36.
[8] Yokoyama, Y. Clinico-pathological studies on the evolution of large bowel carcinoma. *J. Med. Soc. Nagoya* 1990; **40**: 301–319.
[9] Igari, T., Tei, S., Iwasaki, Y. *et al.* Growth pattern and macroscopic features of colorectal cancer. *Stomac. Intest.* 1993; **28**: 1199–1207.
[10] Arima, N., Toyonaga, J., Tsuruta, O. *et al.* Endoscopic diagnosis of the invasion depth of early colonic cancers. *Endoscop. Digest.* 1992; **4**: 1333–1342.
[11] Hirata, I., Moriyama, H., Sugimoto, K. *et al.* An issue concerning endoscopic diagnosis of superficial colorectal epithelial neoplasm. *Stomac. Intest.* 1992; **27**: 903–909.
[12] Tada, S., Iida, M., Yao, T. *et al.* Stereomicroscopic findings of adenoma and early cancer in the colorectum. *Stomac. Intest.* 1992; **27**: 949–961.
[13] Sugahara, A. A scanning electron microscopic study of small superficial adenomas in the colon with special reference to depressed changes. *J. Med. Soc. Kawasaki* 1995; **21**: 1–13.
[14] Kawai, S. A new sub-classification of superficial colorectal tumor and early cancer by magnifying colonoscopy. *J. Jpn. Soc. Colorectol.* 1994; **47**: 485–491.
[15] *General Rules for Clinical and Pathological Studies on Cancer of the Colon, Rectum and Anus.* 6th edn. Japanese Society for Cancer of the Colon and Rectum. 1998.
[16] Jass, J.R. and Sobin, L.H. *WHO Histological Typing of the Intestinal Tumors.* Berlin, Germany: Springer-Verlag, 1989.
[17] Nakamura, K., *Structure of Large Bowel Cancer.* Tokyo Igaku-shoin, 1989.
[18] Kudo, S., Takano, Y., Hayashi, S. *et al.* The clinicopathological features of flat and depressed type of early colorectal cancer. *Stomac. Intest.* 1989; **24**: 317–329.
[19] Fujinuma, S. and Sakai, Y. Clinicopathological characteristics of superficial type colorectal adenomas obtained by endoscopic resection. *Diag. Therap. Endosc.* 1995; **2**: 99–105.
[20] Kudo, S., Miura, K., Takano, Y. *et al.* Detection of colorectal minute cancer. *Stomac. Intest.* 1990; **25**: 801–812.
[21] Muto, T., Kamiya, J., Sawada, T. *et al.* Small "Flat Adenoma" of the large bowel with special reference to its clinicopathologic features. *Dis. Colon Rectum* 1985; **28**: 847–851.
[22] Watari, J., Saito, Y., Orii, Y. *et al.* Prospective observation of superficial colorectal neoplasms. *Stomac. Intest.* 1996; **31**: 1599–1606.
[23] Hirata, I., Morioka, H., Sasaki, S. *et al.* Diagnosis and therapeutic guideline for minute (less than 5 mm) colorectal adenoma – from the viewpoint of its macroscopic appearance and pit pattern. *Stomac. Intest.* 1995; **30**: 1485–1490.
[24] Kudo, S., Kusaka, H., Nakajima, K. *et al.* Minute surface structure of the depressed type early colorectal cancer. *Stomac. Intest.* 1992; **27**: 963–975.
[25] Tsuda, S., Yao, T., Matsui, T. *et al.* Superficial colonic neoplasm diagnosed by endoscopy: comparison of radiologic and morphologic pictures. *Stomac. Intest.* 1992; **27**: 935–947.
[26] Ajioka, Y., Watanabe, H., Chida, T. *et al.* Pathological characteristics of small superficial type epithelial neoplasia of the large intestine with special reference to its macroscopic criteria. *Stomac. Intest.* 1990; **25**: 837–846.
[27] Wada, R., Matsukuma, S., Abe, H. *et al.* Histopathological studies of superficial-type early colorectal carcinoma. *Cancer* 1996; **77**: 44–50.
[28] Hayakawa, M., Shimokawa, K., Kusugami, K. *et al.* Clinicopathological feature of superficial depressed-type colorectal neoplastic lesions. *Am. J. Gastroenterol.* 1999; **94**: 944–949.
[29] Jaramillo, E., Watanabe, M., Slezak, P. *et al.* Flat neoplastic lesions of the colon and rectum detected by high-resolution video endoscopy and chromoscopy. *Gastrointest. Endosc.* 1995; **42**: 114–122.

